# Pathological Significance of Mitochondrial Glycation

**DOI:** 10.1155/2012/843505

**Published:** 2012-06-21

**Authors:** Pamela Boon Li Pun, Michael P. Murphy

**Affiliations:** MRC Mitochondrial Biology Unit, Wellcome Trust/MRC Building, Cambridge CB2 0XY, UK

## Abstract

Glycation, the nonenzymatic glycosylation of biomolecules, is commonly observed in diabetes and ageing. Reactive dicarbonyl species such as methylglyoxal and glyoxal are thought to be major physiological precursors of glycation. Because these dicarbonyls tend to be formed intracellularly, the levels of advanced glycation end products on cellular proteins are higher than on extracellular ones. The formation of glycation adducts within cells can have severe functional consequences such as inhibition of protein activity and promotion of DNA mutations. Although several lines of evidence suggest that there are specific mitochondrial targets of glycation, and mitochondrial dysfunction itself has been implicated in disease and ageing, it is unclear if glycation of biomolecules specifically within mitochondria induces dysfunction and contributes to disease pathology. We discuss here the possibility that mitochondrial glycation contributes to disease, focussing on diabetes, ageing, cancer, and neurodegeneration, and highlight the current limitations in our understanding of the pathological significance of mitochondrial glycation.

## 1. Introduction

Glycation is a common feature of diabetic complications [1, 2] and ageing pathologies [3, 4]. This nonenzymatic glycosylation process stems from multiple reactions between reducing sugars or sugar derivatives and amino groups on proteins, lipids and nucleotides. Glycation involves multiple oxidative and nonoxidative reactions, collectively termed the Maillard reaction, eventually generating advanced glycation end products (AGEs) [5]. In clinical and experimental models of diabetes and ageing, the levels of intra- and extracellular AGEs have been found to increase relative to healthy or young controls [3, 6–10]. Such correlative data suggests that glycation can potentially contribute to disease progression and ageing pathology. However, whether there is a causal relationship between glycation and pathology is uncertain and the mechanistic details are unclear.

**Figure 1:**
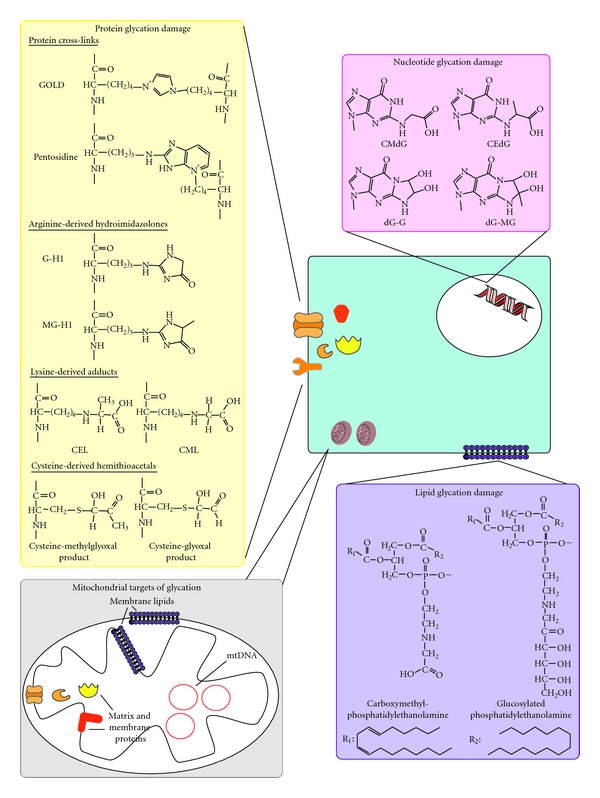
Reactive *α*,*β*-dicarbonyl-induced glycation damage in cells. Glycation by *α*,*β*-dicarbonyls like methylglyoxal and glyoxal affects all classes of biological macromolecules. Proteins, in particular arginine, lysine, and cysteine residues, are highly susceptible to glycation, forming protein cross-links and various AGE adducts, examples of which are shown here. Of these, the most important protein-AGEs quantitatively are arginine-derived hydroimidazolones, especially MG-H1. Formation of protein AGEs alters protein structure and function, leading to biochemical dysfunction. Nucleotides and lipids may also undergo glycation, with deoxyguanosine and basic phospholipids being particularly vulnerable. Consequences of nucleotide and lipid glycation damage are increased DNA mutations and compromised lipid membrane integrity, respectively. Within mitochondria, it is expected that glycation affects matrix and membrane proteins, phospholipids on the outer and inner mitochondrial membranes and mtDNA.

That the formation of AGEs can have significant functional consequences further supports this hypothesis. Glycation affects all major classes of biomolecules (Figure 1), with damage levels estimated at 0.1–1% of lysine and arginine residues on proteins, 1 in 10^7^ nucleotides on DNA and 0.1% of basic phospholipids [11]. The consequences of such glycation damage can be severe. The formation and accumulation of protein AGEs, for instance, can result in biochemical dysfunction. For example, some AGEs like MOLD (methylglyoxal-derived lysine dimers) and pentosidine form protein crosslinks, altering protein structure, generally causing proteins to become more resistant to proteolysis [12]. Protein structure may also be modified as a result of charge neutralization when arginine and lysine residues are glycated [13]. Consequently, structural integrity of proteins becomes compromised. For instance, glycated collagen is stiff and nonelastic relative to the non-glycated protein [14, 15]. The change in charge distribution could also promote protein aggregation, for instance, of lens crystallins, leading to cataract formation in diabetes and in old age [16, 17]. Changes in protein conformation could also influence its function, as would glycation of amino acid residues at sites for substrate binding and allosteric regulation on enzymes. It has been shown, for instance, that methylglyoxal-induced glycation of lys 126 and arg 463 in glutamate dehydrogenase isolated from bovine liver affects its ability to bind its substrate and allosteric activator (adenosine diphosphate (ADP)), respectively, resulting in a decrease in glutamate dehydrogenase activity [18]. Similarly, glycation of extracellular matrix (ECM) proteins affects cell-ECM interactions. The reaction of methylglyoxal with arginine residues on the RGD and GFOGER motifs in the integrin-binding sites of collagen, for instance, resulted in endothelial cell detachment by interfering with cell-ECM interactions [7]. 

Membrane interactions [19] may also be affected by the formation of lipid glycation adducts which increase membrane fluidity [20]. Lipids possessing a free amino group such as phosphatidylethanolamine are susceptible to glycation, whereas those without, for example phosphatidylcholine, are not glycated [21]. It is thought that lipid glycation may promote lipid peroxidation, resulting in oxidative damage [22–24]. For example, *in vitro* incubation of human low-density lipoprotein (LDL) with 200 mM glucose for up to 12 days increased levels of both glycation and lipid oxidation products, which appears to support the hypothesis that lipid glycation causes, or at least enhances, lipid peroxidation [22]. However, since lipid glycation and peroxidation can both occur in parallel, it is difficult to distinguish one from the other as being the primary initiating event versus a secondary downstream event, particularly *in vivo* [22].

Likewise, glycation of DNA can have multiple effects such as strand breaks, unwinding of the double helix, mutations and formation of DNA-protein and nucleotide-nucleotide cross-links [25–30]. The extent of modification appears to be dependent on the glycating agent used. For example, methylglyoxal induced 10-fold more DNA-protein crosslinks than did glyoxal when incubated *in vitro* with DNA and DNA polymerase I derived from *Escherichia coli* [31]. Such DNA-DNA polymerase crosslinks could stall replication and subsequently promote frameshift mutations [31]. The steric hindrance imposed by DNA glycation adducts may also impair transcription by preventing transcription factors from binding [32]. That is, DNA glycation may not only influence genome integrity but could also alter gene expression. 

Besides direct glycation damage to biomolecules, AGEs, especially extracellular AGEs, could also contribute to disease pathology by binding to cell surface receptors such as the receptor for AGEs (RAGE), thereby activating the proinflammatory NF-*κ*B pathway and downstream signalling molecules such as p21, MAP kinases, and JNK [33, 34]. 

**Figure 2:**
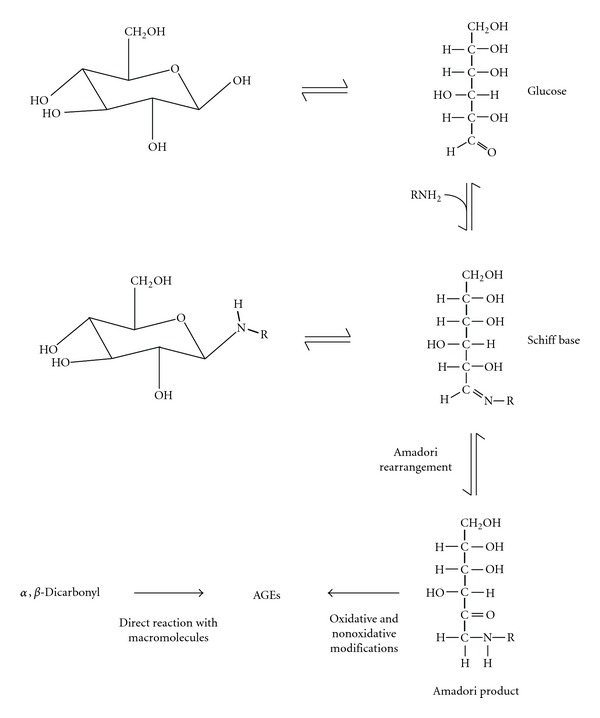
Formation of AGEs in physiological systems. In early glycation, glucose reacts reversibly with amine groups on macromolecules to form a Schiff base and subsequently an Amadori product. This can undergo further oxidative and nonoxidative modifications to form AGEs. Alternatively, AGEs may also be formed by the direct reaction of reactive *α*,*β*-dicarbonyls with macromolecules.

**Figure 3:**
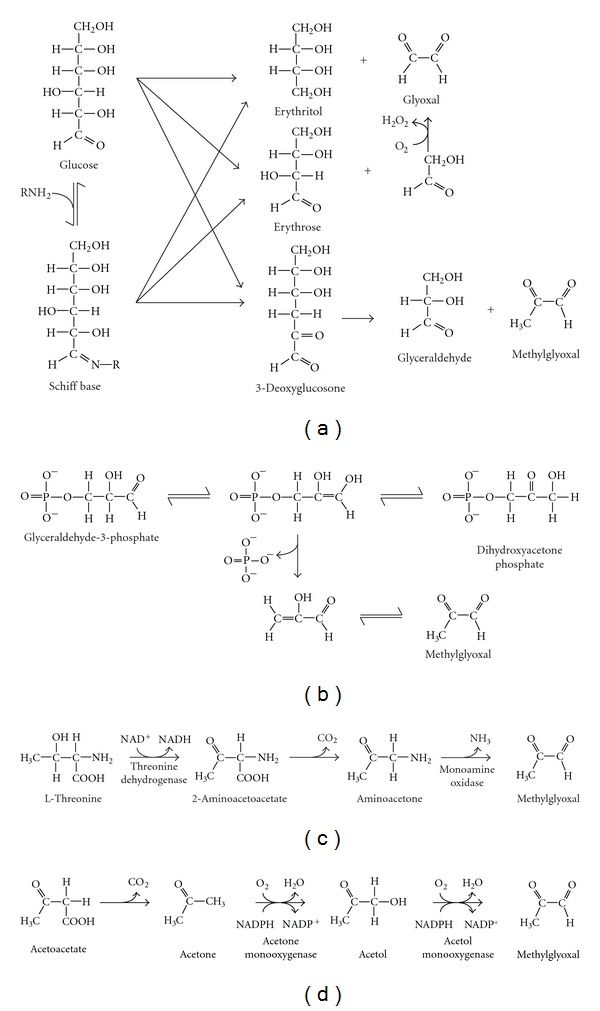
Formation of methylglyoxal and glyoxal in biological systems. (a) The acyclic form of glucose may undergo autooxidation via the Wolff pathway to form glyoxal or break down to 3-deoxyglucosone which in turn degrades to glyceraldehyde and methylglyoxal. Glucose may also react with amine groups on proteins to form a Schiff base which can undergo similar breakdown via the Namiki pathway to also generate glyoxal and methylglyoxal. (b) The major pathway by which methylglyoxal is formed in cells involves the breakdown of the triose phosphates, glyceraldehyde-3-phosphate, and dihydroxyacetone phosphate, via phosphate elimination from an ene-diol intermediate. (c) Methylglyoxal may also be generated during threonine catabolism. This involves oxidation of threonine by threonine dehydrogenase to 2-aminoacetoacetate, followed by spontaneous decarboxylation to aminoacetone. Monoamine oxidase then catalyses the conversion of aminoacetone to methylglyoxal. (d) Another pathway by which methylglyoxal is produced is from acetoacetate metabolism. This reaction proceeds via acetone and acetol and is catalysed by acetone monooxygenase and acetol monooxygenase.

The formation of AGEs arises from reactions between reducing sugars (e.g., glucose) or sugar derivatives (e.g. *α*,*β*-dicarbonyls) and amino groups on biological macromolecules (Figure 2). Glycation reactions can be broadly classified as early and late stages of glycation. During early glycation, the carbonyl group of acyclic glucose reacts with amino groups, then undergoes dehydration to form a Schiff base, which subsequently undergoes further rearrangement to form a more stable Amadori product, fructosamine [35] (Figure 2). Alternatively, the Schiff base may undergo spontaneous degradation to form reactive *α*,*β*-dicarbonyl species such as methylglyoxal and glyoxal [36] (Figure 3(a)). These dicarbonyls may also be formed from glucose breakdown via the Wolff pathway [37] (Figure 3(a)), triose phosphate fragmentation [38] (Figure 3(b)), acetone [39] and threonine metabolism [40] (Figures 3(c) and 3(d)), lipid peroxidation [41], and fructose-3-phosphate decomposition [42]. Downstream of Amadori product formation, further oxidative and nonoxidative modifications may occur, generating AGEs [5] (Figure 2). Where oxidative processes are involved, the term glycoxidation has also been used [43]. AGEs can also be formed by the direct modification of amino groups by *α*,*β*-dicarbonyls (Figure 2).

Although glycation is traditionally thought of as a reaction between sugars and amino groups, other functional groups on macromolecules, for example, protein thiols (-SH), may also react in analogous reactions [44]. Similar to glycation of lysine and arginine residues on proteins, glycation of thiol groups can modulate enzyme activity. For example, glyceraldehyde-3-phosphate dehydrogenase can be inhibited by methylglyoxal-mediated loss of thiol groups at its active site [44]. Glycation of creatine kinase by glyoxal also led to enzyme inactivation [45]. Although thiol modification by *α*,*β*-dicarbonyls could be damaging to protein function, low molecular weight thiols such as N-acetylcysteine and GSH have been suggested as therapeutic agents for the removal of *α*,*β*-dicarbonyls before they can react with proteins [45, 46]. 

Despite the common association of glycation with glucose, this reducing sugar is not itself highly reactive. Rather, it is reactive *α*,*β*-dicarbonyl species such as methylglyoxal and glyoxal, which are up to 50,000-fold more reactive than glucose, that are thought to be the major physiological precursors of glycation damage [47]. Because these dicarbonyls are mostly produced intracellularly through processes such as triose phosphate fragmentation [38] and lipid peroxidation [41], there is a greater likelihood for cellular proteins to be glycated relative to plasma proteins. Therefore, levels of AGEs tend to be higher in cellular proteins than in plasma proteins. For instance, levels of the methylglyoxal/arginine-derived hydroimidazolone, MG-H1 [N^*δ*^-(5-hydro-5-methyl-4-imidazolon-2-yl)ornithine], were found to be 1.22 mmol/mol arginine in human blood cells and 0.92 mmol/mol arginine in plasma proteins [48]. Similarly, levels of another AGE, CML (N^*ε*^-carboxymethyl-lysine), was at least three-fold higher in cellular proteins (0.068–0.233 mmol/mol lysine) than in plasma proteins (0.021 mmol/mol lysine) [48]. 

**Figure 4:**
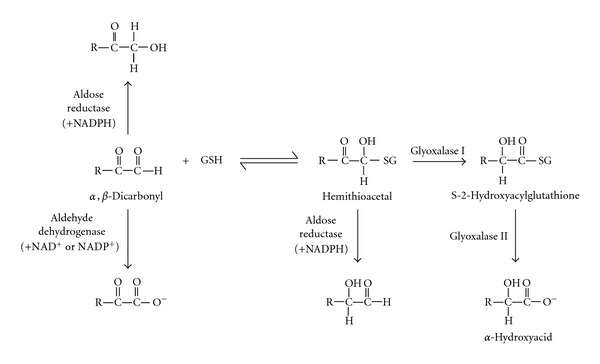
Physiological defences against *α*,*β*-dicarbonyl precursors of glycation. *α*,*β*-dicarbonyls like methylglyoxal (R:CH_3_) and glyoxal (R:H) can be removed by aldose reductase-catalysed reduction to the corresponding alcohol or by oxidation to pyruvate and glycolate. respectively, catalysed by aldehyde dehydrogenase. Reactive *α*,*β*-dicarbonyls may also combine reversibly with reduced glutathione to form a hemithioacetal which can then be reduced by aldose reductases, forming lactaldehyde and glycoaldehyde from methylglyoxal and glyoxal, respectively. Alternatively, the hemithioacetal may undergo a two-step conversion to an *α*-hydroxyacid, catalysed by glyoxalase I and glyoxalase II. The glyoxalase enzyme system represents the major pathway by which methylglyoxal is removed.

To minimize glycation damage by reactive *α*,*β*-dicarbonyls, there exist various enzyme systems that catalytically remove these species (Figure 4). One of the most important of these defences is the glyoxalase enzyme system which functions primarily to remove methylglyoxal and, to a lesser extent, other compounds such as glyoxal [49, 50]. Using glutathione (GSH) as a cofactor, glyoxalase I catalyzes the formation of *S*-2-hydroxyacylglutathione from methylglyoxal or glyoxal. Glyoxalase II then converts this intermediate compound into an *α*-hydroxyacid (either D-lactate from methylglyoxal or glycolate from glyoxal), regenerating GSH in the process. Other enzymes involved in the detoxification of *α*,*β*-dicarbonyls include aldehyde dehydrogenases which oxidize methylglyoxal and glyoxal to pyruvate and glyoxylate, respectively [51], and aldo-keto reductases/aldose reductases which reduce them to form alcohols (e.g., methylglyoxal to acetol and lactaldehyde, glyoxal to glycoaldehyde and ethylene glycol) [52]. 

Under normal physiological conditions, these anti-glycation defences are sufficient to prevent significant glycation damage. Any damage that does occur is dealt with by damage repair and removal systems. For example, proteasomes and lysosomes prevent the accumulation of glycated proteins [53]; the nucleotide excision repair system removes glycated nucleotides [54]; lipid turnover helps clear glycated lipids [19]. When there is an imbalance between the production of glycation precursors such as *α*,*β*-dicarbonyls and the removal of these species such that the former is favoured, carbonyl stress occurs [55] and glycation damage accumulates.

Because *α*,*β*-dicarbonyls are the major precursors of AGEs and their production is largely intracellular, it is likely that there would be significant functional consequences of AGEs formation and accumulation at the organellar level. In this review, we focus on glycation in mitochondria and its association with mitochondrial dysfunction in disease and ageing.

## 2. Glycation in Mitochondria: Involvement in Diabetes and Ageing

The levels of both intra- and extracellular AGEs have been found to increase in diabetic or old animals relative to healthy or young controls [3, 6–10]. Indeed, a glycation adduct of glucose to haemoglobin, HbA_1c_, is used clinically as an indicator of glycaemic control [56, 57], and levels of glycation adducts like CML and pentosidine have been suggested as prognostic tools in diabetics [58]. The accumulation of AGEs arises from an increase in their production and/or a decrease in their removal. For instance, low protein turnover is responsible for the accumulation of extracellular AGEs on collagen with age [59]; in diabetics, plasma levels of HbA_1c_ increase as a result of high levels of blood glucose which forms an adduct to the N-terminal valine on the *β*-chain of haemoglobin [56, 57]. 

**Figure 5:**
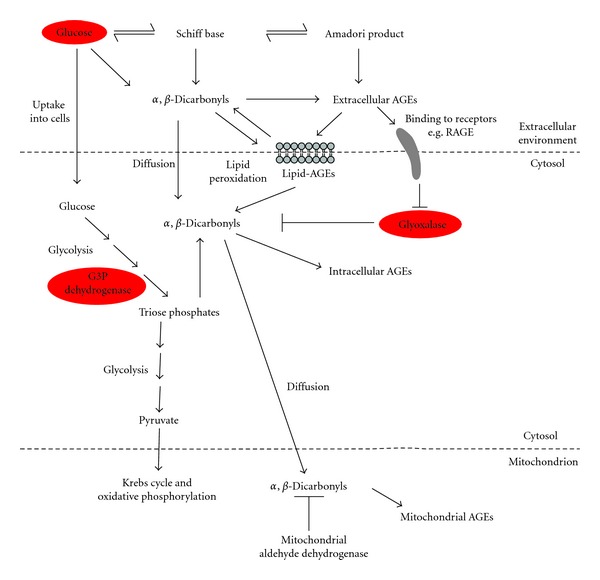
Intra- versus extracellular glycation—involvement in diabetes and ageing. Glycation can take place in the extracellular environment or within cells in the cytosol and in organelles like mitochondria. Extracellular AGEs may arise from oxidative and nonoxidative modifications of the Amadori product or from direct reaction of *α*,*β*-dicarbonyls with proteins. Extracellular AGEs may bind to cell surface receptors such as RAGE, thereby activating cell signalling pathways. The formation of lipid-AGEs on the cell surface membrane can further generate reactive *α*,*β*-dicarbonyls such as glyoxal. These reactive carbonyl species can diffuse through lipid membranes and enter cells where they react with cellular biomolecules to form intracellular AGEs. They can also diffuse further into mitochondria and similarly cause glycation damage within these organelles. Intracellular glycation may also arise from *α*,*β*-dicarbonyls produced during the breakdown of triose phosphates generated during glycolysis. Normally, glycation damage is kept under control by defences such as the glyoxalase enzyme system and aldehyde dehydrogenases. However, in diabetes, glycation damage increases due to elevated formation of *α*,*β*-dicarbonyls, arising from high glucose levels and consequent inhibition of the glycolytic enzyme, G3P dehydrogenase, both of which lead to a rise in triose phosphate levels. These in turn break down nonenzymatically to form methylglyoxal, a major precursor of glycation. In addition, activation of RAGE is associated with decreased glyoxalase I expression, which is expected to further raise methylglyoxal levels by preventing its removal. During ageing, glycation damage also increases, but mainly as a result of a loss of glyoxalase activity with age.

Within cells, the levels of AGEs also increase with age or in diabetes, and this is likely to reflect changes in intracellular *α*,*β*-dicarbonyl levels (Figure 5). In the case of diabetes, the levels of intracellular AGEs rise extremely quickly compared to that of extracellular AGEs and are thought to be a result of the intracellular formation of the reactive dicarbonyl species, methylglyoxal and glyoxal [60]. According to the unifying hypothesis proposed by Brownlee [1], mitochondrial superoxide production increases during hyperglycaemia, resulting in DNA damage and poly(ADP-ribose) polymerase (PARP) activation. ADP-ribose polymers generated by PARP may then bind glyceraldehyde-3-phosphate (G3P) dehydrogenase, inhibiting it. Consequently, there is a build-up of glycolytic intermediates upstream of this enzyme. Among these intermediates are the triose phosphates, G3P and dihydroxyacetone phosphate (DHAP), which break down to form methylglyoxal [38]. In addition, oxidative stress resulting from hyperglycaemia promotes lipid peroxidation [61, 62], another source of a major *α*,*β*-dicarbonyl species, glyoxal [41]. As such, there is increased formation of reactive dicarbonyls in diabetes. With respect to ageing, decreases in glyoxalase I activity have been reported in *Caenorhabditis elegans* [3] and rats [63]. The less efficient removal of *α*,*β*-dicarbonyls consequently leads to elevation of the levels of AGEs in older animals [3]. 

Once formed within the cell, these reactive dicarbonyl species may then diffuse across membranes [47, 64] and access mitochondrial targets (Figure 5). It is estimated that there is 1-2 mmol CML/mol lysine in rat heart mitochondria [65], 0.5–1 mmol CML/mol lysine in rat liver mitochondria [66] and 0.7–1 mmol CML/mol lysine in rat brain mitochondria [67]. Corresponding values for carboxyethyl-lysine (CEL) are 0.5, 0.2–0.5, and 0.1–0.2 mmol CEL/mol lysine [65–67]. It is striking that levels of CML and CEL in mitochondria from the three tissues (heart, liver, and brain) are kept within narrow range (<2 mmol AGE/mol lysine). This suggests that excessive glycation of mitochondrial proteins is highly damaging and that levels of mitochondrial AGEs have to be minimized to prevent mitochondrial dysfunction. Likewise, mitochondrial DNA (mtDNA) [68] and lipids [69] are also targets for glycation. Indeed, measurements of CEdG [N2-(1-carboxyethylguanine], a nucleotide glycation adduct, were found up to three-fold more CEdG in mtDNA of cultured fibroblasts than in nuclear DNA [68], suggesting that mtDNA is more vulnerable to glycation than nuclear DNA due to the absence of protective histones in the former [70]. The presence of glycated phosphatidylethanolamine in mitochondrial membranes has also prompted suggestions that mitochondrial lipid glycation could affect electron transport chain activity and cause mitochondrial dysfunction [69]. 

Intriguingly, specific mitochondrial protein targets of glycation have been identified in kidneys of streptozotocin-induced diabetic rats [71] and in livers of aged rats [18]. Increasing the removal of *α*,*β*-dicarbonyls either by administering the prototypical dicarbonyl scavenger, aminoguanidine, or by overexpression of glyoxalase I decreased both glycation and oxidative damage, restored complex III activity, and improved respiration in experimental models of diabetes [71, 72]. Likewise, increasing glyoxalase I expression in nematodes decreased mitochondrial levels of methylglyoxal and AGEs, and extended lifespan, while inhibiting the enzyme increased methylglyoxal levels and reduced lifespan [3]. These results suggest that glycation of mitochondrial proteins could account, at least in part, for the mitochondrial dysfunction and oxidative damage observed in hyperglycaemia and ageing. Separately, changes in the expression of mitochondrial proteins have been observed in Schwann cells grown under hyperglycaemic conditions [73] and in Akita mice, a mouse model of type 1 diabetes [74]. While the molecular mechanisms inducing these changes have not been elucidated, one study in cultured endothelial cells found that glycation could cause epigenetic changes in expression of nuclear-encoded genes [75], highlighting the potential for glycation to do likewise in mitochondrial genes, thereby prompting the “remodelling of the mitochondrial proteome” as is observed in Akita mice [74]. 

The hypothesis that glycation of mitochondrial targets profoundly influences mitochondrial function is supported by studies in which exogenous dicarbonyls were administered and mitochondrial parameters studied. For example, methylglyoxal treatment of isolated mitochondria from rat kidney [76] and from several carcinoma cell lines [77–79] decreased oxygen consumption by mitochondria. Similarly, treatment of cultured cells with methylglyoxal or glyoxal decreased mitochondrial membrane potential reduced the activities of the respiratory chain complexes, reduced ATP synthesis, and increased reactive oxygen species (ROS) levels [80–83]. While these studies all point towards a glycation-induced mitochondrial dysfunction, the physiological relevance of such experiments is uncertain. Some have suggested that the use of millimolar concentrations of dicarbonyls in such experiments is physiologically irrelevant [47, 84], especially since cellular levels of methylglyoxal have been estimated to be in the low micromolar range. Others, however, have argued that these values, being measures of steady state concentrations of dicarbonyls, do not accurately reflect the actual dicarbonyl flux within cells where *α*,*β*-dicarbonyls are produced and that millimolar concentrations are reasonable estimates of the actual flux *in vivo* [85]. That only a very small proportion of exogenous dicarbonyl becomes incorporated into cells has also been used to justify the use of high concentrations of exogenous dicarbonyls [83, 86]. It should also be noted that in treating cultured cells with exogenous methylglyoxal or glyoxal, extracellular, cytosolic and mitochondrial levels of these dicarbonyls and their associated AGEs are all increased. Therefore it is unclear whether any mitochondrial dysfunction observed is a direct result of the glycation of mitochondrial targets or if it is a downstream consequence following the glycation of extracellular or cytosolic targets especially since incubation of cells with exogenous AGEs can similarly induce mitochondrial dysfunction [87–89].

## 3. Mitochondrial Glycation in Cancer and Neurodegeneration: A Hypothesis

As Experimental uncertainties notwithstanding, the issue of mitochondrial glycation and its links to disease is scientifically and clinically interesting. Should mitochondrial glycation be a major cause of disease, then a broad strategy of limiting glycation damage in mitochondria may ameliorate disease initiation and progression across multiple clinical conditions. Such a strategy would be akin to that of targeting antioxidants to mitochondria for the treatment of conditions as disparate as Parkinson's disease, diabetes and ischaemia-reperfusion injury [90]. In this context, it is intriguing to consider whether mitochondrial glycation could contribute to conditions apart from diabetes, and ageing. We discuss here the possibility of this hypothesis in cancer and neurodegeneration. 

### 3.1. Cancer

The Warburg phenotype in which aerobic tumour cells are largely dependent on glycolysis, rather than oxidative phosphorylation, for their energy supply has been well-described in many cancer cell lines [91]. Since triose phosphate intermediates of the glycolytic pathway are an important source of the major glycation precursor, methylglyoxal [38], the levels of methylglyoxal and consequently glycation should be increased in tumour cells. It appears that the type and distribution of such glycation damage varies between tumour types, and not all tumours necessarily display equal extents of glycation [92]. In addition, overexpression of glyoxalase I and glyoxalase II has been correlated with multidrug resistance in tumours [93]. The upregulation of the glyoxalase enzyme system is thought to allow tumour cell growth by counteracting the rise in methylglyoxal production [93]. However, tumour cells with high glyoxalase I expression have higher levels of DNA glycation adducts than those with relatively lower expression [94] suggesting that glyoxalase I expression increases to try to counteract methylglyoxal-induced cytotoxicity [94]. It is therefore not surprising that glyoxalase I inhibitors are being evaluated for use in cancer chemotherapy [95] as they render multidrug-resistant tumours vulnerable to apoptosis [96]. Glyoxalase I inhibitors may potentiate apoptosis by increasing DNA glycation that results in PARP activation which depletes cellular nicotinamide adenine dinucleotide (NAD^+^) and thereby inhibits G3P dehydrogenase, leading to further increases in methylglyoxal formation [97]. Methylglyoxal also induces cytotoxicity in a variety of other ways such as promoting the mitochondrial permeability transition pore, activation of protein kinase C-delta [93] and inhibition of STAT3-associated signaling [98]. 

As in diabetes and ageing, mitochondria are profoundly affected in cancers; the upregulation of glycolysis in tumour cells is accompanied by mitochondrial dysfunction and decreased oxidative phosphorylation [99, 100]. For example, a recent review [101] argues that such mitochondrial dysfunction is critical for tumour growth, citing a strong correlation between tumour progression and increased mitochondrial DNA (mtDNA) mutations. Although the role of mitochondrial glycation in cancers has not been explored, it is possible that elevated glycolysis in cancer cells leads to increased glycation and mutation of mtDNA [75]. Thus up-regulation of glycolysis in tumour cells may contribute to increased mitochondrial damage and thereby establish a vicious cycle that enhances the Warburg effect. 

### 3.2. Neurodegeneration

As triose phosphates are a major source of methylglyoxal [38], any disturbance in triose phosphate metabolism will influence methylglyoxal formation. For example, triose phosphate isomerase (TPI) deficiency results in an inherited neurological disorder [102]. This occurs because TPI catalyzes the interconversion of DHAP and G3P, and its inhibition results in an accumulation of DHAP which breaks down to methylglyoxal [103]. Methylglyoxal-induced protein and nucleotide glycation might result in paralysis and neurodegeneration as is observed in flies expressing a mutant form of TPI [104]. Nitrotyrosinated TPI has been detected in brains from mouse models of, and human patients with, Alzheimer's disease [105], and this nitration of TPI decreases its enzyme activity and increases methylglyoxal production [105]. Tau aggregation and neurofibrillary tangle formation can also be promoted by methylglyoxal [106, 107]. These suggest that methylglyoxal and glycation can contribute to the progression of neurodegeneration, and this is supported by observations that glyoxalase I expression increases in the brains of early and middle stage Alzheimer's patients [108], indicating an attempt to scavenge excessive *α*,*β*-dicarbonyls. That type II diabetics are at 2–2.5 fold greater risk of developing dementia also lends weight to a contribution of glycation in neurodegenerative diseases [109]. It has also been proposed that intra- and extracellular AGEs contribute to neurodegeneration by two main pathways, the former by promoting protein aggregation and thus inhibiting their proper function, and the latter by accumulating on senile plaques and inducing oxidative stress and inflammation [110]. 

The role of mitochondrial dysfunction in neurological disorders has been extensively reviewed [111–113]. While mitochondrial glycation was not specifically investigated in these studies, it may be that glycation damage of proteins involved in oxidative phosphorylation and of mtDNA may contribute to the mitochondrial dysfunction observed. Specific protein targets of carbonylation, oxidation, and nitration have been identified in mitochondria in neurodegenerative disorders [114, 115]. Since glycation and oxidative damage are closely correlated, with both types of damage markers often increasing in parallel with ageing and disease [22–24], it is plausible that glycation of specific mitochondrial targets also occurs. A recent study in a mouse model of Alzheimer's disease further found that mitochondrial dysfunction precedes the presentation of any neurodegenerative pathology [116]. Particularly striking was the observation in the same study of increased glycolysis and decreased oxidative phosphorylation in neurons from these mice, features reminiscent of the Warburg phenotype of tumour cells. The many similarities between the conditions discussed above—diabetes, ageing, and cancer—with neurodegeneration suggest a potential for mitochondrial glycation to contribute to these conditions. 

## 4. Conclusion

In the four conditions discussed above—hyperglycaemia, ageing, cancer, and neurodegeneration—there is increased production of glycation precursors, namely, reactive *α*,*β*-dicarbonyls such as methylglyoxal and glyoxal, leading to elevated molecular glycation damage as evidenced by the rise in levels of AGEs. However, despite the correlation between glycation, mitochondrial dysfunction, and disease, the relative importance of mitochondrial glycation as opposed to extracellular or cytosolic glycation is still unclear. This is in part because it is the whole cell levels of *α*,*β*-dicarbonyls that are altered by experimental manipulation with aminoguanidine or glyoxalase I overexpression, and also because treatment of cell cultures with exogenous methylglyoxal or glyoxal raises both extra- and intracellular dicarbonyl levels. As such, the effects of any changes are not isolated to mitochondria. Besides, the amount of exogenous dicarbonyl that is physiologically relevant is a further point of contention. Unfortunately, it is technically difficult to accurately measure dicarbonyl levels within mitochondria in cells and *in vivo* as existing methods are prone to artefacts arising from variations in mitochondria isolation, sample preparation, and derivatization [84, 117, 118]. There is also a lack of experimental tools for manipulating dicarbonyl levels within mitochondria alone without modifying cytosolic and extracellular levels. Therefore, it is difficult to isolate the effects of glycation observed in mitochondria from more general consequences on the whole cell. Consequently, to divorce the contribution of mitochondrial glycation from cytosolic or extracellular glycation to disease is experimentally challenging. Nonetheless, the prospect of mitochondrial glycation contributing as a common damaging agent across a broad spectrum of diseases is an intriguing possibility and is also a novel potential therapeutic target.

## Abbreviations

ADP: Adenosine diphosphate
AGEs: Advanced glycation end products
ATP: Adenosine triphosphate
CEdG: N2-(1-carboxyethylguanine)
CEL: Carboxyethyl-lysine
CML: N^*ε*^-carboxymethyl-lysine
DHAP: Dihydroxyacetone phosphate
ECM: Extracellular matrix
G3P: Glyceraldehyde-3-phosphate
GSH: Reduced glutathione
LDL: Low-density lipoprotein
MG-H1: [N^*δ*^-(5-hydro-5-methyl-4-imidazolon-2-yl)ornithine]
MOLD: Methylglyoxal-derived lysine dimers
mtDNA: Mitochondrial DNA
NAD^+^: Nicotinamide adenine dinucleotide
PARP: Poly(ADP-ribose) polymerase
RAGE: Receptor for AGEs
ROS: Reactive oxygen species
TPI: Triose phosphate isomerase.
